# Moderator Band and Ventricular Tachycardia: Structural or Functional Substrate?

**DOI:** 10.3390/jcdd10040159

**Published:** 2023-04-06

**Authors:** Federico Landra, Carmine Marallo, Amato Santoro, Simone Taddeucci, Maria Cristina Tavera, Claudia Baiocchi, Alberto Palazzuoli

**Affiliations:** 1 Division of Cardiology, Department of Medical Biotechnologies, University of Siena, 53100 Siena, Italy; f.landra@student.unisi.it (F.L.); carmine.marallo@gmail.com (C.M.); simone.taddeucci@gmail.com (S.T.); 2 Division of Cardiology, Cardio Thoracic and Vascular Department, Azienda Ospedaliera Universitaria Senese, 53100 Siena, Italy; amato.santoro@gmail.com (A.S.);; 3 Cardiovascular Diseases Unit, Cardio Thoracic and Vascular Department, Azienda Ospedaliera Universitaria Senese, University of Siena, 53100 Siena, Italy; mariacristina.tavera@ao-siena.toscana.it

**Keywords:** ventricular tachycardia, electrical disorder, moderator band, Purkinje fibers, ablation, pharmacological treatment

## Abstract

The moderator band (MB) is an intracavitary structure of the right ventricle composed of muscular fibers encompassing specialized Purkinje fibers, separated each other by collagen and adipose tissue. In the last decades, premature ventricular complexes originating within the Purkinje network have been implicated in the genesis of life-threatening arrhythmias. However, right Purkinje network arrhythmias have been much less reported in the literature compared to the left counterpart. The MB has unique anatomical and electrophysiological properties, which may account for its arrhythmogenicity and may be responsible for a significant portion of idiopathic ventricular fibrillation. MB embodies autonomic nervous system cells, with important implications in arrhythmogenesis. Some idiopathic ventricular arrhythmias, defined as the absence of any identifiable structural heart disorder, can begin from this site. Due to these complex structural and functional peculiarities strictly interplayed each other, it is arduous to determine the precise mechanism underlying MB arrhythmias. MB-related arrhythmias should be differentiated from other right Purkinje fibers arrhythmias because of the opportunity for intervention and the unusual site for the ablation poorly described in the literature. In the current paper, we report the characteristics and electrical properties of the MB, their involvement in arrhythmogenesis, clinical and electrophysiological peculiarities of MB-related arrhythmias, and current treatment options.

## 1. Introduction

Moderator band (MB) related ventricular tachycardias (VTs) and premature ventricular contractions (PVC) are being increasingly recognized as a cause of idiopathic VTs. The MB is a muscular structure extending from the anterior septum to the anterior papillary muscle (APM) of the right ventricular (RV) free wall. This structure comprises a specialized Purkinje fibers network which allows for rapid activation of the RV-free wall [1]. The MB is composed of muscular fibers encompassing specialized Purkinje fibers, separated from the surrounding muscular cells by collagen and adipose tissue. Additionally, it is highly innervated by autonomic nervous system elements [2], with important implications in arrhythmogenesis. Idiopathic ventricular arrhythmias, defined by the absence of any identifiable structural heart disease or genetic arrhythmic disorder, have usually been considered “benign” in terms of prognosis [3]. Nonetheless, in the last decades, short coupling PVCs [4] e and PVCs originating within the Purkinje network have been described as a trigger of life-threatening arrhythmias and characterization and treatment of such idiopathic ventricular arrhythmias have become increasingly investigated [5,6,7]. After the development of sophisticated imaging tools such as intracardiac echocardiography (ICE) and intracardiac mapping, MB has been increasingly recognized as a source of short-coupling PVCs, VTs, and idiopathic ventricular fibrillation (IVF); [8,9]. The arrhythmogenic potential of MB involves multiple mechanisms due to the peculiar anatomical and electrical properties of this structure. Purkinje fibers may initiate arrhythmias by different mechanisms, mainly enhanced automaticity, trigger activity, and re-entry [10]. Sustained VTs are instead supported by a reentrant mechanism, favored by the action potential duration (APD) gradient established between MB and RV, and can be treated by catheter ablation [11].

## 2. Anatomy of the Moderator Band

The MB is an intracavitary structure of the RV which spans from the interventricular septum (IVS) to the RV-free wall at the base of the APM, and it is considered part of the septomarginal trabeculation. The MB has high interindividual variability in terms of origin and shape [12]. It might originate from the supraventricular crest to the ventricular apex, with single or double roots. Moreover, it can be classified into three major types according to its shape: cylindrical column (type 1), long and thin column (type 2), and wide and flat column (type 3) [13]. The most common configuration of MB is a single root origin from the middle IVS with a type 2 shape.

Originally described by Leonardo Da Vinci, the MB takes its name from what it was thought to be its function, which is to prevent RV excessive distension thanks to the anatomic link of the IVS and RV free wall. Later, it was found that part of the right half of the Purkinje system is contained in the MB, allowing for synchronous activation of the RV-free wall during systole. The right Purkinje system is composed of the right bundle branch (RBB), the free-running Purkinje fibers (PF), which branch from the RBB along its length, and the terminal PF, which connects with the free-running PF in the parietal RV wall. The terminal PF account for the largest part of the right Purkinje network, forming a more complex network than that in the left ventricle that cover the majority of the RV free wall and extends toward the tricuspid valve [12]. PF spreads across the IVS through the MB, becomes more superficial in the distal third of the MB, and finally joins the APM to the RV-free wall, forming the subendocardial ventricular plexus.

PF are a longitudinal assembly of Purkinje cells (PC) and are insulated from working myocytes by a connective tissue sheath that is lost before the PF form the terminal connections with the working myocytes through specialized junctions in the endocardium [14,15]. Recently, Walton et al. [11] demonstrated that the MB has a compartmentalized structure between the myocardium and PF, which have a coaxial configuration separated each other by a layer of collagen and lipid droplets that provide electrical insulation. Therefore, the MB is formed by these two excitable yet electrically uncoupled compartments.

The MB vasculature is perfused by the anterior interventricular coronary artery and the right coronary artery from the base of the APM, forming an important collateral circulation between the left and right coronary artery systems [1].

Innervation of the MB is supplied by elements of the autonomic system, being the PF more sensible to the parasympathetic neurotransmitter acetylcholine (ACh) compared to ventricular muscle because of a higher expression of channels responsible for I_KACh_ current [2,16].

## 3. Electrical Properties of the Moderator Band

PC are specialized myocardial cells that are responsible for the synchronous activation of the ventricles. Ion channel expression substantially differs from that of the working myocites and can explain the specialized electrical activity of the PC [12]. Differently from working myocardium, PC are poorly contractile and, therefore, have a lower expression of Ca^2+^-handling proteins such as RYR2 (Ryanodine receptor 2), RYR3 (Ryanodine receptor 3), SERCA2a (sarcoplasmatic/endoplasmic reticulum Ca^2^ ATPase 2a), and NCX (Na^+^-Ca^2+^ exchangers). In contrast, they are fast conducting because of high upstroke velocity during phase 0 of action potential and high conduction velocity. These properties are explained by a higher expression of Na^+^ channels, particularly Na1.1, and of the fast-conducting connexin Cx40. Regarding the K^+^ channel profile, PC show a lower expression of Kv1.4 and a higher expression of Kv4.2, the first being a slow recovering isoform while the second being a fast one, both responsible for I_to_ (transient outward current). On the other hand, the K^+^ conductance involved in phase 2 and phase 3, namely I_Ks_, I_Kr_, and I_Kur_ (for whom KvLQT1, ERG, and Kv1.5 are responsible, respectively), are predicted to be lower in PC as compared to working myocardium. In addition, the I_K1_ current generated by the K_ir_2 channels family (K_ir_2.1–2.4) responsible for the maintaining of resting potential is less represented in PC, and HCN 1 and HCN4, which underlie I_f_ current, are more expressed. All these differences account for the following differences in action potential: high upstroke velocity during phase 0, a prominent early rapid repolarization (phase 1), a negative potential plateau (phase 2), an increased APD, and a spontaneous diastolic depolarization (phase 4), which is normally inapparent because of overdrive suppression by the sinus node. Additionally, it has been shown that genetically determined upregulation of the DPP6 (dipeptidyl aminopeptidase-like protein 6) gene, which encodes for a beta subunit of Kv4 channels, enhances I_to_ in PF with consequent strong repolarization gradient between the PF and the adjacent ventricular myocardium, with possible phase 2 re-entry induced PVCs [17].

**Figure 1:**
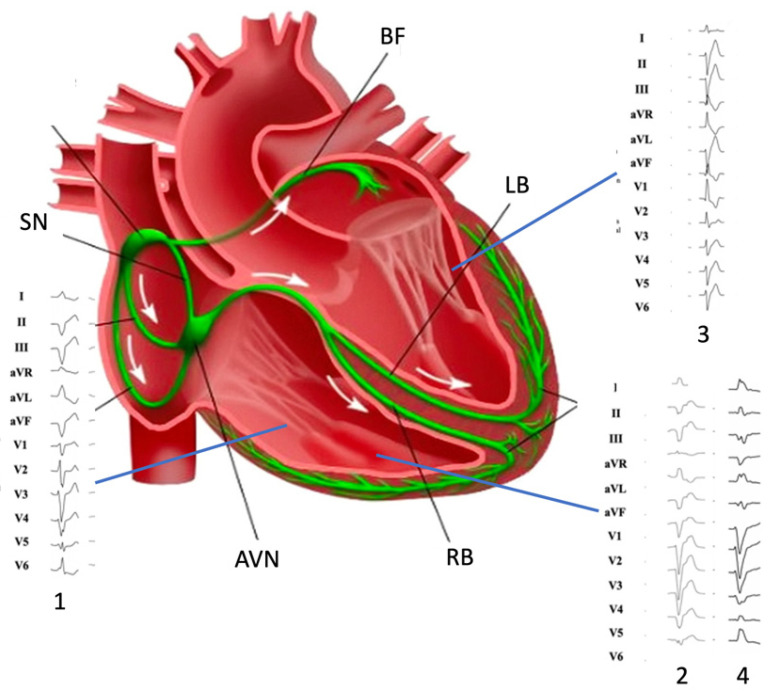
Morphology of premature ventricular complexes originating from (1) inferior tricuspid valve, (2) moderator band, (3) left posterior fascicle, and (4) moderator band (note DII/DIII discordance). SN: Sinus Node; AVN: atrio-ventricular node; RB right bundle; LB: left bundle; BF: Bachmann’s fibers.

Moreover, the electrophysiological properties of MB may be influenced by its anatomic variability [11]. Particularly, variability of thickness and muscle/PFs ratio may be associated with a reduction of the effective refractory period, which is sensitive to tissue mass and coupling. Many factors may as well affect the dispersion of refractoriness within the myocardial syncytium represented by the MB, such as mechanical stretch (i.e., RV overload, bradycardia, conditions that induce a prolongation of APD; [18] and cardiac autonomic nervous system modulation [19]. These features may explain the ECG morphologies of PVC (Figure 1).

## 4. Moderator Band-Related Arrhythmias

### 4.1. Arrhythmogenic Mechanisms

All arrhythmogenic mechanisms may be involved in the genesis of MB-related arrhythmias, particularly triggered activity and re-entry. PF are often involved in the genesis of ventricular arrhythmias due to their susceptibility to the development of early afterdepolarizations (EADs). EADs seem to be ascribable mainly to inward calcium currents (L-type Ca^2+^) but also to late Na^+^ currents during late phase 2/early phase 3 of the action potential. Moreover, PVCs may originate from a phase 2 re-entry because of the strong repolarization gradient between the two compartments of the MB [20,21]. A critical number of cells exhibiting afterdepolarizations in a synchronous manner is essential to generate arrhythmias at the organ dimension. The susceptibility of the RV-free wall-MB junction to generate short-coupling PVCs may be related to the mechanical strain of the MB during proto-diastole, a “mechanoelectrical feedback” phenomenon that may induce ion channel activation [22].

Short-coupled PVCs originating in the proximal MB may determine a unidirectional conduction block within the myocardial compartment of the MB and consequently induce a sustained monomorphic VT through a macro re-entry circuit. The Purkinje network must overcome the large source-sink mismatch where it couples with the myocardium. The high density of Na^+^ channels is essential to provide sufficient safety factors for conduction. Moreover, a transitional layer of cells serves to amplify impulses and shield the Purkinje network from electrotonic loading. The wavefront propagates from the IVS to the RV-free wall and then activates it both in basal and apical directions. While the apical wavefront collides at the MB root with the repolarization wave tail, the basal propagation front encounters excitable tissues of the IVS, sufficiently delayed to allow the circuit completion. The demonstration of the involvement of these structures in the re-entry circuit has been provided by the experiment conducted by Walton et al. that showed the interruption of the macro reentry circuit after severing the MB in a sheep heart during sustained VT [11].

Additionally, polymorphic VT, like torsades de pointes, may result from a meandering or drifting spiral wave propagation. Both monomorphic and polymorphic VT, when an electrical or structural obstacle is encountered, may eventually degenerate into ventricular fibrillation (VF) because multiple spiral waves break up with the consequent genesis of daughter spirals. MB-related VF still represents a rare but not neglectable percentage of IVF [23]. Some case series described a VT and VF related to early coupled PVC originating from the MB [8]. Short coupled torsade de point (sc-TdP) should be classified as idiopathic VF from PF.

Furthermore, 3D-mapping and intracardiac echocardiography showed that sc-TdP predominantly originated from MB free wall and its PF network [24].

### 4.2. Right Purkinje Network Arrhythmias: In Addition to the Moderator Band

As mentioned, the right Purkinje system is composed of the RBB, free-running PF, and terminal PF, which form a complex network at the RV free wall extending up to the tricuspid valve (TV); [12]. The electrophysiological properties of the PC, which intrinsically justify a proarrhythmic behavior, may theoretically result in arrhythmias originating at each level of the complex right Purkinje network. However, right Purkinje network arrhythmias and their potential deleterious consequences have been less reported in the literature. Therefore, MB-related arrhythmias should be differentiated from other right-sided PF arrhythmias, such as those from APM and TV. VTs originating at the APM level seem to be provokable by isoproterenol or burst pacing, suggesting triggered activity as the underlying mechanism [25]. Moreover, the inability to entrain VTs from APM or terminate them by overdrive pacing supports automaticity instead of re-entry as a potential inducible mechanism.

The electrocardiographic (ECG) analysis may help clinicians in hypothesizing these specific arrhythmias. MB-related arrhythmias are typically classified among those with a superior axis configuration, such as those from inferior TV and left posterior fascicles [26,27,28] (Figure 1). Typically, MB-related arrhythmias have a left superior axis (positive D1 and negative DII/DIII) and late precordial transition (later than V_4_), similar to those originating from APM [29]. Sometimes, DII/DIII discordance may be observed, such as for lateral TV and para-Hissian origins. TV arrhythmias may display a variable precordial transition depending on the more septal or lateral origin (from V_2_ to V_5_) for those with a superior axis. On the contrary, left posterior fascicle arrhythmias usually mimic a typical RBB pattern with rsR’ and specifically have a QRS < 130 ms because of the rapid ventricular depolarization through the Purkinje system.

Sometimes monomorphic VTs may sustain through a re-entry circuit involving both the right and left bundle branches (called bundle branch re-entry), a single left fascicle (intrafascicular re-entry), or both left fascicles (interfascicular re-entry). However, the intrafascicular re-entry has not been demonstrated at the right Purkinje system level yet. Bundle branch re-entry VTs are often observed in patients with acquired heart disease, which might involve the His-Purkinje system, and, therefore, would lead to slow conduction within the Purkinje system necessary for the occurrence of re-entry. However, conduction disturbances in the His-Purkinje system of young patients with structurally normal hearts can also lead to susceptibility to bundle branch reentry VTs [30]. Finally, MB could be involved in atrioventricular reciprocating arrhythmias due to the insertion of an accessory pathway (Mahaim fascicles) into the PF of the MB.

## 5. Diagnostic of Moderator Band Related Tachycardia

### 5.1. Initial Evaluation 

Patients may have a high variability of clinical presentation, ranging from isolated PVCs, either symptomatic or asymptomatic, to electrical storm and cardiac arrest. Most of the cases of MB-related arrhythmias have been described in middle-aged males. The actual prevalence of these arrhythmias is unknown because of a lack of reports in the literature, and described cases are usually those who have experienced a major event such as cardiac arrest [9].

The initial evaluation of patients presenting with an arrhythmic event does follow the generic algorithm proposed by the latest ESC guidelines on ventricular arrhythmias and sudden cardiac death until a specific diagnosis is reached [23]. Patients with symptomatic or asymptomatic (incidental finding) PVCs/non-sustained ventricular tachycardia (NSVT) should undergo a thorough evaluation which starts from PVCs/NSVT analysis (morphology, coupling interval, burden, cycle length in case of NSVT, relation with physical stress), and covers personal history (cardiovascular risk factors, symptoms such as palpitations, dyspnea, angina and syncope, comorbidities and medications), family history (sudden cardiac death, primary electrical disease, cardiomyopathy), and laboratory tests. In case of arrhythmic syncope, fast monomorphic NSVT, or short coupled PVC followed by polymorphic NSVT, the patients should be admitted to the hospital, and subsequent evaluation performed during hospital admission; otherwise, an ambulatory evaluation is sufficient [23]. On the other hand, patients presenting with a first episode of sustained ventricular tachycardia (SVT) should be similarly evaluated, but hospital admission is mandatory because of the risk of major arrhythmic events. In these cases, the ECG analysis could be particularly helpful in determining the site of origin of PVC/NSVT and, as mentioned above, MB-related arrhythmias usually display a left superior axis (positive D1 and negative DII/DIII) and late precordial transition (later than V_4_), but may also present with a DII/DIII discordance [4,9].

On the contrary, in patients presenting with a cardiac arrest, usually, a complete ECG documentation of the arrhythmic event is unavailable; hence, it may initially remain unknown the involvement of the MB in the genesis of PVC triggers. Therefore, those patients who survive the event should be evaluated with the aim of excluding the ischemic etiology or other structural heart diseases, primary electrical diseases, metabolic or toxic causes, and extracardiac causes through brain and chest CT scan [17,26].

### 5.2. Diagnostic Tests

First-line testing for patients presenting with PVCs/NSVT/SVT includes a 12-lead ECG, a complete echocardiogram, and at least 24 h continuous ECG monitoring (Holter ECG in ambulatory setting). When there is suspicion of an underlying coronary artery disease or structural heart disease, coronary angiography and/or cardiac magnetic resonance should be performed. Otherwise, if a primary electrical disease is suspected, further evaluation should be considered, such as a genetic test, exercise test, and sodium channel blocker test. However, patients with moderator band-related arrhythmias usually show negative results of the abovementioned examinations; therefore, the arrhythmic events are to be labeled as idiopathic. Symptomatic patients with idiopathic PVCs/VTs could be subsequently referred for catheter ablation or treated with drug therapy, whereas asymptomatic patients with a high burden of PVCs (>10%) should undergo a regular follow-up and optionally be referred for catheter ablation [23,26].

Similarly, sudden cardiac arrest survivors in whom all diagnostic examinations have failed to provide a diagnosis should be labeled as IVF. In this case, it is important not to miss the opportunity to perform comprehensive clinical testing (sudden cardiac death family history, personal medical history, ECG, exercise test, and echocardiogram) of first-degree family members to prevent sudden cardiac death. In patients who have experienced IVF, an ICD implantation is mandatory, but the involvement of the MB in the genesis of PVCs that may trigger VF can remain ignored until further events occur or catheter ablation of PVC triggers is considered [31].

## 6. Management of Moderator-Band Ventricular Tachycardias

### 6.1. Ablation Targets

**Figure 2:**
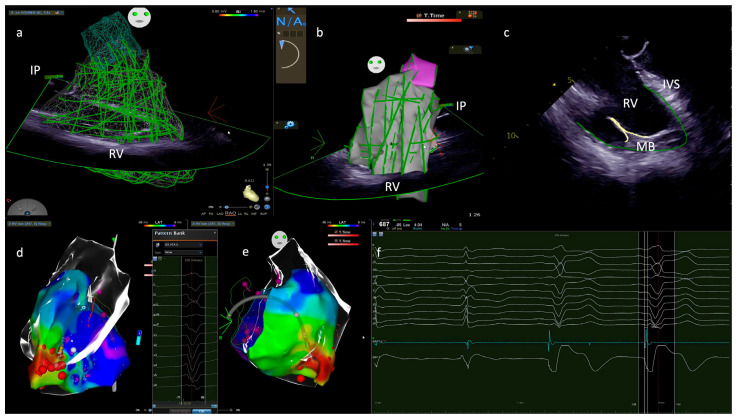
(**a**) Intracardiac echocardiography view; IP is in the right atrium in the right anterior oblique view; green lines paint the long and short axis of RV, constructing a 3D RV echo–map. (**b**) same image in the left anterior oblique view; (**c**) two−dimensional view of RV. The green lines trace the endocardial surface, and yellow lines the MB; (**d**) Merge of the 3D echo map and activation map of PVC in lateral view; (**e**) PVC activation map in right anterior oblique view; ablation at the earliest activation site on the MB using an electroanatomic mapping system; the red color on the activation map is the earliest activation site; the red bullets are the RF lesion tagged points; (**f**) electrogram PVC potential; sharp and QS unipolar signal and adequate PVC anticipation. IP: intracardiac probe; RV: right ventricle; MB: moderator band; 3D: three dimensional; PVC: premature ventricular complex; RF: radiofrequency.

**Figure 3:**
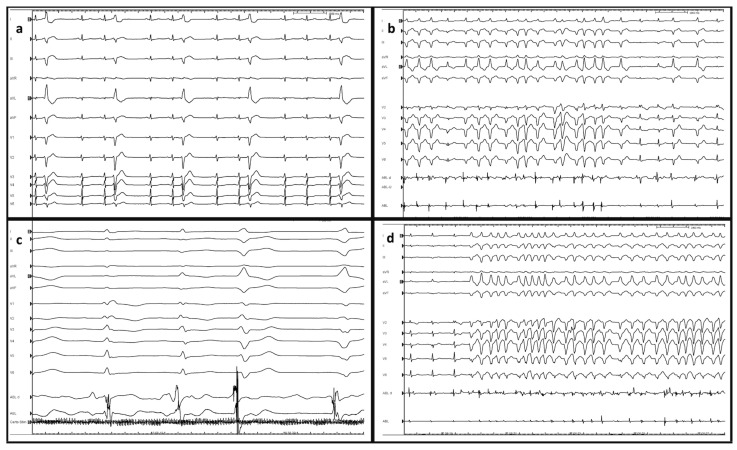
(**a**) Basal Electrogram and PVC; (**b**) Ventricular tachycardia; (**c**) Identification of Purkinje fiber potential during catheter ablation of premature ventricular complex originating from moderator band. Purkinje fiber potential is represented by a sharp, short-lasting, high-frequency potential and prepotential; (**d**) ventricular tachycardia during RF.

Radiofrequency (RF) catheter ablation seems to be an effective approach in the treatment of MB-related arrhythmias. Several case reports have shown good results in suppressing PVCs-mediated VF and electrical storms [8,9]. An electro-anatomic map merged with intracardiac ultrasound images is fundamental to correctly identify, localize and treat arrhythmic foci located in the right Purkinje network (Figure 2). PF potential, represented by a sharp, short-lasting, high-frequency potential, is often the earliest potential recorded before the onset of the PVCs (Figure 3). Ablation performed targeting PF potentials was often successful in suppressing PVCs-mediated VF. The most frequent site of origin of VF/VT trigger was the left-sided PF, followed by the outflow tract region and MB. The role of PF in the genesis of VT/VF has been well described, with the targeting of the earliest pre-QRS PF potential [8].

The anatomic variability of the MB could account for multiple PF-mediated PVCs morphology. Therefore, multiple sites of ablation, of which the whole MB, its insertion in the RV free wall and IVS and the APM, have to be considered in order to completely eliminate the PVCs source, even though it has been reported that ablation at the earliest PF potential recorded is generally effective in suppression of PVCs aside from their morphology [32,33].

Complete ablation of the MB and of its insertion points in the right ventricle is fundamental also to interrupt the macro re-entry circuit. Using contact force-sensing catheters with vector analysis in addition to ICE is fundamental to guarantee sufficient contact and stability of the catheter tip to the ablation site, as well as using higher RF energy. Cryoablation may configure a therapeutic option to achieve arrhythmic substrate disappearance and effective MB ablation [8,31].

### 6.2. Pharmacological Therapy

There is a lack of strong evidence concerning the pharmacological management of MB-related arrhythmias. Nonetheless, since PF are the most likely trigger for arrhythmias, it seems reasonable to manage them as usual for idiopathic VF. Quinidine has been reported to be effective in suppressing PVCs arising from the Purkinje network, considering its ability to suppress the I_to_. Haissaguerre and colleagues demonstrated that repetitive activity from the distal PF network may cause VF even with minimal or no PVCs at baseline. Quinidine is a class Ia sodium channel blocker that also reduces the potassium currents IK1 and IKto. This combined effect may make quinidine uniquely effective in controlling arrhythmias associated with action potential duration dispersion and phase 2 reentry, postulated in IVF. Particularly, Ito is highly expressed in PF, possibly explaining the unique effect of quinidine. Several studies have demonstrated a reduced incidence of ventricular arrhythmias in patients with IVF treated with quinidine. The transient outward potassium current Ito is highly active in the Purkinje network of the MB, and its inhibition may be the mechanism by which quinidine has a therapeutic effect in these arrhythmias. It must be considered that the effective dose of quinidine may be poorly tolerated and should be cautiously tested in each patient [3,8]. Moreover, lidocaine, propafenone, and amiodarone could be considered in these cases, even though they are rarely effective if administered before catheter ablation [9]. Radiofrequency ablation remains a good therapy that has been shown to effectively suppress Purkinje fiber-triggered ectopy and IVF [34].

Over the past 10 years, some reports have described insufficient pharmacological treatment due to the failure of amiodarone, b-blockers, Magnesium, lidocaine, quinidine, cilostazol, and verapamil [9]. Medical therapy for MB-related VTs is limited. Quinidine may be an effective medical therapy in PVC after catheter ablation. The ablation of PVCs at MB-RV free wall junction led to excellent short- and long-term results, and it should probably be considered as the first-line therapy in these patients to reduce VTs mediated from PF at MB level [32].

### 6.3. Device Therapy

In cases of sustained VTs/IVF causing cardiac arrest, ICD implantation in secondary prevention is mandatory [23]. Nevertheless, it should be considered to perform catheter ablation and/or administer appropriate medical therapy to reduce or eliminate the number of ICD shocks. There is a lack of reports concerning ICD implantation in primary prevention before and after unsuccessful catheter ablation [33]. The prognosis of MB-related VT/VF without any therapy is uncertain. We do suggest that an invasive therapeutic strategy could be positively performed to prevent malignant arrhythmias and cardiac arrest. It is of valuable importance to consider the long-term impact that ICD implantation may carry in these patients since they are usually middle-aged, particularly when for primary prevention. Indeed, they may experience device-related complications and undergo various generator changes during their lifetime. Subcutaneous ICD (s-ICD) should be preferred because of the absence of a need for pacing. In addition, psychological impact deriving from ICD carrying and possibly from shock occurrence, either appropriate or inappropriate, should be considered as well [35]. For these reasons, the ablation strategy gains an even stronger rationale over device therapy.

### 6.4. Sports Eligibility and Risk Stratification

**Figure 4:**
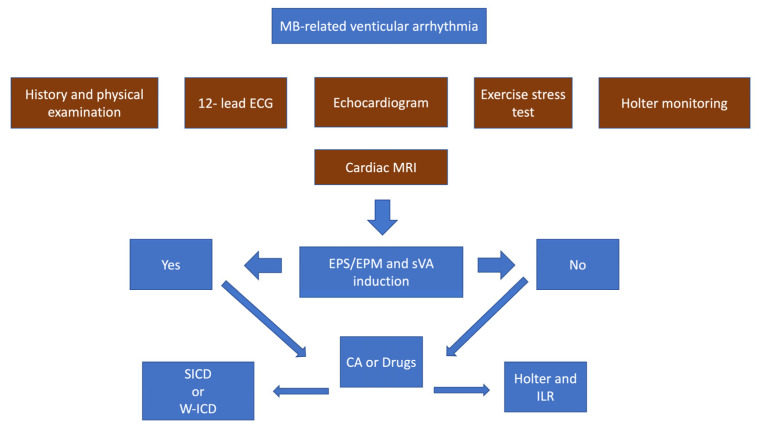
Comprehensive list of examinations and therapeutic strategies available for the management of patients with MB-related ventricular arrhythmias. EPS: electrophysiological study; electrophysiological mapping; sVA: sustained ventricular tachycardia; SICD: subcutaneous implantable defibrillator; W-ICD: wearable ICD; ILR: implantable loop recorder; CA: catheter ablation.

There are no specific indications for patients presenting with MB-related arrhythmias regarding lifestyle modification and sport participation. Recommendations for these patients follow those of the latest guidelines on sports cardiology and exercise in patients with cardiovascular diseases [36]. Recommendations are dependent on the clinical presentation of the patient, which has been shown to be highly variable. Therefore, patients with isolated PVCs and NSVT are permitted to participate in competitive and leisure-time sports activities with periodic re-evaluation based on Holter monitoring, 12-lead ECG, exercise test, and echocardiography. High-burden PVCs and NSVT have not been correlated with unfavorable outcomes, even in an asymptomatic pediatric population and without structural heart disease [37]. Continuation of intensive or competitive sports participation in patients with malignant arrhythmias or already implanted with an ICD should undergo shared decision-making, considering the absence of underlying structural disease at risk of progression and the absence of proven correlation of arrhythmias occurrence concomitantly with sports activities. In this setting, non-invasive evaluation could provide unclear results during the sports eligibility assessment. Recently, it has been proposed a comprehensive invasive workflow for the assessment of patients presenting with ventricular arrhythmias [38]. Particularly, electrophysiological study (EPS) and electroanatomical (EAM) mapping could provide important information for risk stratification during the sports eligibility assessment. However, no specific protocol has been reported for the electrophysiological procedures in this context so far. It is assumed that patients with positive EPS for ventricular arrhythmias inducibility or abnormal EAM have a higher risk for sudden cardiac death than those who show negative results at EPS and EAM. In the first case, a catheter ablation procedure and/or s-ICD implantation could be planned. In the meanwhile, it could be reasonable to protect the patient with a temporary solution such as a wearable ICD (w-ICD). On the contrary, patients with negative electrophysiological assessment could undergo only monitoring, ideally with loop recorder implantation, as shown in Figure 4.

### 6.5. Follow-Up

Patients with asymptomatic PVC/NSVT who have not been referred for a specific treatment should be regularly followed-up, but no specific time lapse between visits has been suggested from recent guidelines. It is reasonable to perform a complete ambulatory visit comprehensive of echocardiogram and a Holter ECG each 6 to 12 months in order to monitor left ventricular ejection fraction for the exclusion of PVC-induced cardiomyopathy and evaluate arrhythmias’ burden. In case of the development of PVC-induced cardiomyopathy or an elevated burden of arrhythmias, specific treatment must be considered, and catheter ablation represents a valid therapeutic option.

Patients who have undergone a specific drug treatment or catheter ablation should be equally followed-up in order to evaluate the efficacy of the chosen treatment, both in terms of symptoms and reduction/suppression of the arrhythmic burden and to exclude possible adverse events. For example, quinidine assumption may lead to overdose, which may present with symptoms linked to the involvement of the central nervous system (i.e., dizziness, headache, nausea, vomiting, hearing, and visual symptoms) or cardiac side effects such as conduction disturbances and proarrhythmic effect. Of note, in case of quinidine overdose, it could be helpful to acidify the patient’s urine to favor excretion.

Finally, patients who presented with a cardiac arrest due to IVF and who have undergone an ICD implantation should be regularly followed up to evaluate arrhythmic relapses and assess the delivery of ICD therapies such as ATP and shocks. For this purpose, it is of particular importance the implementation of a program of remote monitoring, which may promptly lead to an appropriate treatment. In case of recurrent ICD shocks, it is possible to implement a pharmacological therapy, if not present, and perform catheter ablation of the arrhythmic substrate. To conclude, survivors of a cardiac arrest due to IVF could also be referred for genetic counseling, which may identify a potential genetic predisposition to the development of arrhythmias, therefore, help in the prevention process of relatives of the proband.

## 7. Conclusions

The moderator band is an intracavity structure of the right ventricle with unique anatomical and electrophysiological properties. In fact, the moderator band is composed of two distinct compartments of working myocytes and Purkinje fibers presenting a coaxial configuration, both excitable but electrically uncoupled, because of collagen and lipid droplet insulation. Moreover, they functionally distinguish each other by virtue of different ion channels expression, susceptibility to autonomic system modulation, and mechanical stretch influence, which finally translate into action potential duration gradients and repolarization dispersion. Hence, the conduction system compartment of the moderator band displays an intrinsic dynamic proarrhythmic behavior that needs the muscular compartment in order to initiate ventricular tachycardias. In the setting of the moderator band, structure and function are therefore difficultly discernible, and they ideally resemble two faces of the same coin.

## Data Availability

Not applicable.
